# Liver ChIP-seq analysis in FGF19-treated mice reveals SHP as a global transcriptional partner of SREBP-2

**DOI:** 10.1186/s13059-015-0835-6

**Published:** 2015-12-04

**Authors:** Young-Chae Kim, Sangwon Byun, Yang Zhang, Sunmi Seok, Byron Kemper, Jian Ma, Jongsook Kim Kemper

**Affiliations:** Department of Molecular and Integrative Physiology, University of Illinois at Urbana-Champaign, Urbana, IL 61801 USA; Department of Bioengineering, University of Illinois at Urbana-Champaign, Urbana, IL 61801 USA; Carl R. Woese Institute for Genomic Biology, University of Illinois at Urbana-Champaign, Urbana, IL 61801 USA

**Keywords:** FGF15, Bile acid, Nuclear receptor, HMGCR, Cholesterol biosynthesis

## Abstract

**Background:**

Fibroblast growth factor-19 (FGF19) is an intestinal hormone that mediates postprandial metabolic responses in the liver. The unusual orphan nuclear receptor, small heterodimer partner (SHP), acts as a co-repressor for many transcriptional factors and has been implicated in diverse biological pathways including FGF19-mediated repression of bile acid synthesis. To explore global functions of SHP in mediating FGF19 action, we identify genome-wide SHP binding sites in hepatic chromatin in mice treated with vehicle or FGF19 by ChIP-seq analysis.

**Results:**

The overall pattern of SHP binding sites between these two groups is similar, but SHP binding is enhanced at the sites by addition of FGF19. SHP binding is detected preferentially in promoter regions that are enriched in motifs for unexpected non-nuclear receptors. We observe global co-localization of SHP sites with published sites for SREBP-2, a master transcriptional activator of cholesterol biosynthesis. FGF19 increases functional interaction between endogenous SHP and SREBP-2 and inhibits SREBP-2 target genes, and these effects were blunted in SHP-knockout mice. Furthermore, FGF19-induced phosphorylation of SHP at Thr-55 is shown to be important for its functional interaction with SREBP-2 and reduction of liver/serum cholesterol levels.

**Conclusion:**

This study reveals SHP as a global transcriptional partner of SREBP-2 in regulation of sterol biosynthetic gene networks and provides a potential mechanism for cholesterol-lowering action of FGF19.

**Electronic supplementary material:**

The online version of this article (doi:10.1186/s13059-015-0835-6) contains supplementary material, which is available to authorized users.

## Background

Small heterodimer partner (SHP, NR0B2) is an unusual orphan nuclear receptor that lacks a DNA binding domain and acts as a co-repressor for many other nuclear receptors involved in diverse biological pathways [1–4]. Recent studies using genetic mouse models have shown that SHP has a functional role in regulation of lipid/glucose metabolism, circadian control of metabolism, reproduction, and inflammation [5–9]. Of known SHP functions, the role of SHP in feedback repression of bile acid (BA) biosynthesis has been most intensively studied [5, 6, 10]. An endogenous ligand for SHP has not been identified, but protein levels and repression activity of SHP are modulated by post-translational modifications (PTMs) in response to BA or fibroblast growth factor-19 (FGF19) signaling [11–13]. FGF19 (FGF15 in mice) is an intestinal hormone that is induced by the BA-activated nuclear receptor FXR in response to a meal and strongly represses BA synthetic genes in a SHP-dependent manner [14, 15]. Intriguingly, the metabolic action of FGF19 is similar to insulin but FGF19 does not stimulate hepatic lipogenesis [15]. FGF19 also has cholesterol-lowering effects [16], but the underlying mechanisms are not known.

Sterol regulatory element-binding proteins (SREBPs) are a family of basic-helix-loop-helix transcription factors that regulate lipid metabolism [17]. Of the three mammalian isoforms, SREBP-1c mediates insulin-dependent activation of fatty acid synthesis, SREBP-2 is a master transcriptional activator of cholesterol biosynthesis, and SREBP-1a activates both fat and sterol biosynthetic gene programs [18, 19]. SREBP precursors are retained in the endoplasmic reticulum in a complex with INSIG and sterol-sensing SCAP. When cellular sterol levels are low, the complex dissociates and SREBP/SCAP are transported to the Golgi apparatus, SREBP is proteolytically cleaved, and the N-terminal SREBP fragment translocates to the nucleus and activates expression of fat and cholesterol biosynthetic genes [17]. A global role of SREBP-2 in lipid metabolism, as well as a new function, autophagy of lipid droplets under sterol-depleted conditions, was demonstrated by ChIP-seq studies [20].

In this study, we have identified genome-wide hepatic binding sites of SHP by ChIP-seq analysis in mice treated with vehicle or FGF19. SHP binding strikingly overlapped globally with SREBP-2 binding, published previously [20], at many sterol biosynthetic genes, including the HMG CoA Reductase (*Hmgcr*) gene. Utilizing molecular/biochemical approaches and SHP-knockout (KO) mice [5], we further show that treatment with FGF19 or feeding, which induces intestinal synthesis of FGF15 in mice, increases both the functional interaction of endogenous SHP with SREBP-2 in hepatocytes and the inhibition of sterol biosynthesis-related genes in a SHP-dependent manner.

## Results

### Identification of genome-wide binding sites of SHP in hepatic chromatin in mice

We have identified genome-wide binding sites of SHP in hepatic chromatin by ChIP-seq analysis in mice treated with vehicle or FGF19. The quality of the immunoprecipitation with SHP antibody was first confirmed by standard ChIP to detect binding of SHP at its known target genes, *Cyp7a1*, *Cyp8b1*, *Ntcp*, in WT and SHP-KO mice (Additional file 1: Figure S1a). Samples from three independent ChIP from mouse liver were pooled for the ChIP-seq sequencing (Additional file 1: Figure S1b).

**Fig. 1:**
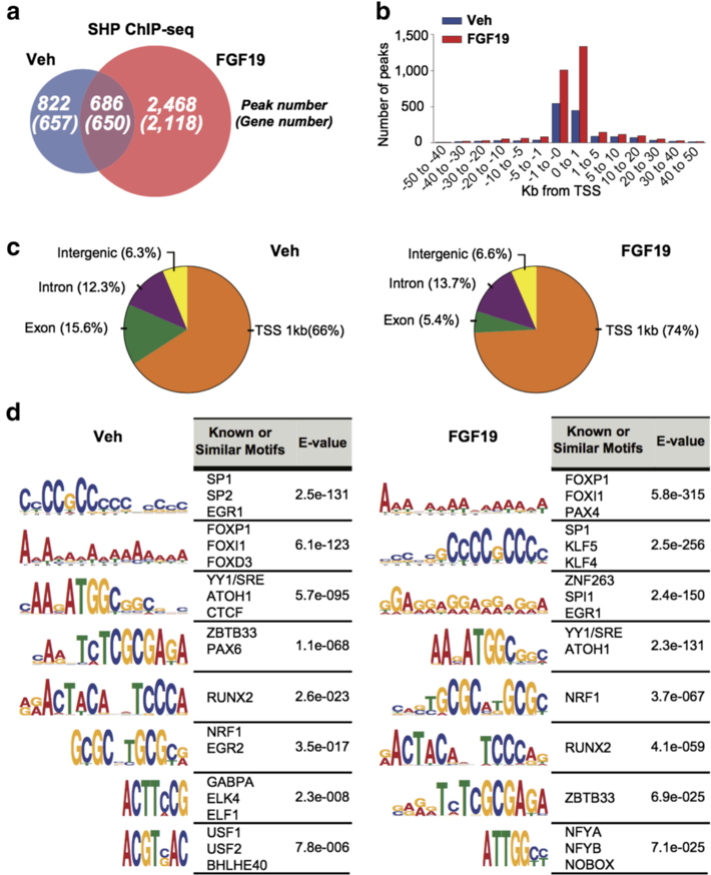
Genomic distribution of hepatic SHP binding and motif analysis of SHP binding regions. **a** Venn diagrams showing the numbers of SHP binding peaks in hepatic chromatin and nearby genes (parentheses) in mice treated with vehicle or FGF19 for 2 h. **b** Distances from the center of SHP binding peaks to the TSS. **c** Genomic distribution of SHP binding peaks in the vehicle- or FGF19-treated group. **d** Logos showing the common sequences detected with highest confidence (E-value) at the SHP binding peak regions. For each common sequence motif, up to the top three transcription factors that potentially bind to the motif are shown

A total of 1,508 and 3,154 peaks were detected in the vehicle-treated and FGF19 samples, respectively, with 686 peaks (at least 1 bp overlap) common to both, and 1,307 and 2,768 genes nearest to the peaks within 50 Kb were identified for the vehicle- and FGF19-treated samples, respectively, with 650 genes common to both (Fig. 1a). Notably, SHP binding peaks were preferentially in the promoter regions with 66 % and 74 % of peaks within 1 kb of transcription start sites (TSS) in mice treated with vehicle and FGF19, respectively (Fig. 1b, c). This analysis revealed a strong preference of SHP binding in the proximal promoter regions of potential SHP target genes in both groups.

### *De novo* motif analysis of SHP binding peak regions

To identify possible transcription factors that recruit SHP to its sites, DNA motifs were detected by *de novo* motif analysis within the SHP binding peak regions (+/- 100 bp of peak summit). The transcription factor motifs for previously unknown SHP partners, such as, SP1, YY1/SREBP-2, EGR1, NF-Y, RUNX2, and NRF1, were identified within SHP binding peaks (Fig. 1d). Since these transcription factors commonly bind to proximal promoter regions, further studies will be required to determine if these factors functionally interact with SHP or are coincidentally located within SHP binding areas.

**Fig. 2:**
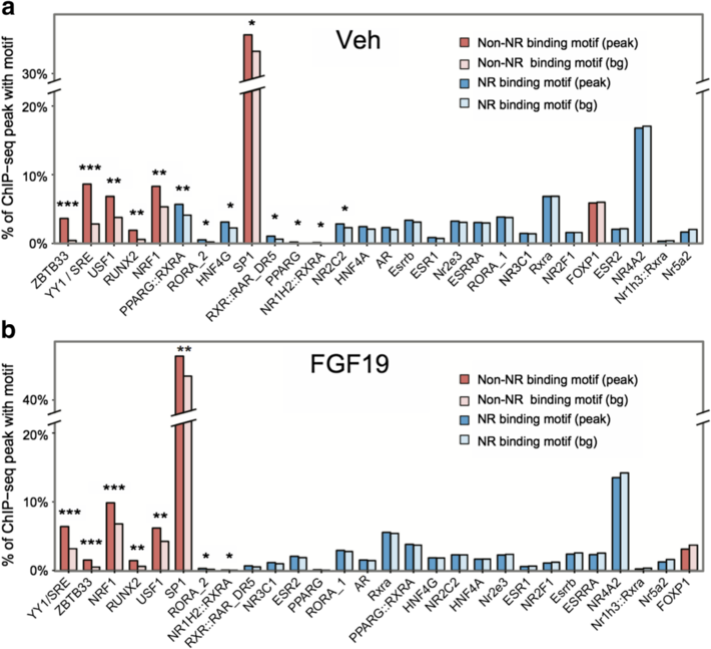
Motif enrichment in peak regions versus randomly selected background sequences. **a**, **b** For each transcription factor, the percentage of SHP peak regions in vehicle (Veh)- (a) or FGF19-treated samples (b) with motifs within +/-100 bp of the peak summits (peak) or the percentage in randomly selected sequences (bg) is shown. Randomly selected sequences are GC normalized to the peak region sequences. ‘Non-nuclear receptor (non-NR)’ binding motifs are for factors that are not in the nuclear receptor superfamily and ‘nuclear receptor (NR)’ motifs are all known nuclear receptor motifs listed in the JASPAR database. Motifs are ordered on the X-axis from smallest *P* value to largest *P* value from left to right based on the binomial test. ***P <1e-10, **P <0.01, *P <0.1. Note that with exception of PPARγ/RXRα in vehicle-treated sample, none of the nuclear receptor motifs has a *P* value smaller than 0.01

SHP has been shown to directly interact with and inhibit many other nuclear receptors, but binding motifs for known SHP-interacting nuclear receptors, such as HNF-4, LXR, PPARγ, ROR, RAR, NURR, and RXRα, were detected only at lower significance than the unexpected non-nuclear receptor binding motifs in both vehicle- and FGF19-treated groups (Fig. 2). These results suggest that in addition to nuclear receptors, SHP may functionally interact with non-nuclear receptor transcription factors that were not previously recognized as transcriptional partners.

### Functional gene ontology (GO) analysis

**Fig. 3:**
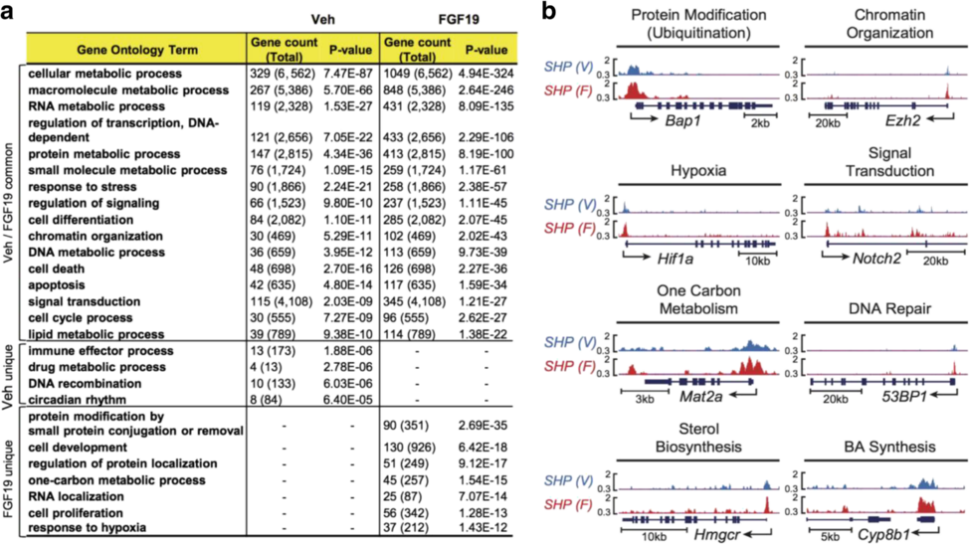
GO analysis and display of SHP binding peaks. **a** Categories (from the top 500 GO annotations) of the potential SHP target genes (nearest to and within 50 Kb of SHP binding peaks using GREAT) detected for both groups and uniquely in each group. **b** Normalized SHP binding peaks at hepatic genes involved in diverse biological pathways are displayed (UCSC genome browser)

To explore biological pathways potentially regulated by SHP, genes nearest to SHP binding peaks were assigned to functional groups by GO analysis. In both groups, the majority of potential SHP target genes were, as expected, present in metabolic pathways (Fig. 3a). In addition to metabolic processes, previously unknown functions were also predicted, which include stress response, signal transduction, chromatin organization, and regulation of transcription. Potential SHP target genes that are uniquely detected in the vehicle-treated sample included genes involved in drug metabolism, the immune process, DNA recombination, and circadian rhythm. Some potential SHP target genes that are uniquely detected in FGF19-treated samples included protein modifications, in particular ubiquitination, developmental process, hypoxia, autophagy, and 1-carbon metabolism (Fig. 3a). Overall, this GO analysis suggests that SHP may have a broader range of biological functions than previously recognized.

Randomly chosen genes with enriched binding in the FGF19 group were analyzed by ChIP to validate the ChIP-seq results. Nearly all of the tested genes, 20 out of 21, showed at least a 1.5-fold increase in SHP binding after FGF19 treatment (Additional file 1: Figure S2). SHP binding peaks detected at hepatic genes involved in selected diverse biological pathways, including BA biosynthesis and transport, are displayed (Fig. 3b, Additional file 1: Figure S3). SHP was shown to be required for FGF15/19-mediated repression of Cyp7a1 [14] but recent tissue-specific genetic mouse studies have indicated that both FGF15 and SHP play important roles in *Cyp8b1* repression, whereas FGF15, but not SHP, preferentially represses *Cyp7a1* gene expression [21]. In line with these recent findings, SHP binding peaks were readily detected at the promoter of *Cyp8b1* gene and relatively smaller peaks were present at the *Cyp7a*1 gene in FGF19-treated sample (Additional file 1: Figure S3). In standard ChIP assays, FGF19 treatment led to more marked binding of SHP to the *Cyp8b1* promoter compared to Cyp7a1 (Additional file 1: Figure S1a).

### Striking global co-occupancy of SHP with SREBP-2 at hepatic genomes

The DNA motif, AAgATGGCggcg, which was detected in SHP peak regions (Fig. 1d), binds YY1 but was also identified as an SREBP-2 motif [20]. In addition, DNA motifs for SP1 and NF-Y that function synergistically with SREBPs [22] were detected in SHP binding peak regions (Fig. 1d). SREBP-2 is a master transcription activator of cholesterol biosynthesis [17], so the possibility that SHP interacts with SREBP-2 to regulate sterol biosynthesis, in addition to its role in sterol catabolism into BAs, was intriguing.

**Fig. 4:**
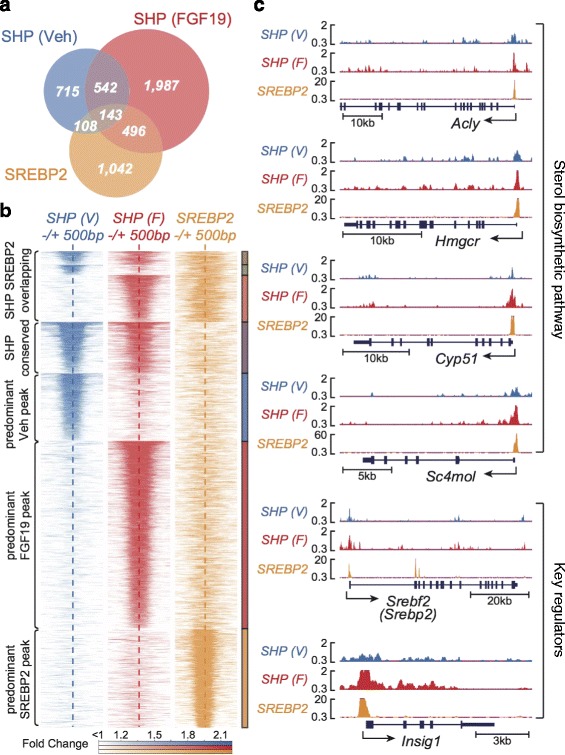
Co-localization of SHP and SREBP-2 binding sites in hepatic genomes. **a** A Venn diagram showing the overlap between the binding peaks of SHP and SREBP-2 [20] in hepatic chromatin. Peaks with at least 1 bp overlap were designated as overlapping. **b** Heat map analysis showing overlapping binding peaks of SHP and SREBP-2 in vehicle- or FGF19-treated group (SHP SREBP2 overlapping); SHP peaks present in both groups (SHP conserved); and peaks present in only one group as indicated (predominant). **c** Normalized ChIP-seq peaks for SHP and SREBP-2 at selected sterol biosynthetic networks are displayed (UCSC genome browser)

Remarkably, about 42 % of the SREBP-2 sites, published previously [20], overlapped with SHP sites (*P* value <2.2e-16, Fisher’s exact test) and about 20 % of the SHP sites overlapped with SREBP-2 sites (Fig. 4a, b). Re-analysis of the sequence data for the published SREBP-2 ChIP-seq study using the same pipeline as used for our SHP CHIP-seq data resulted in about half as many SREBP-2 sites, but a similar percentage of SREBP-2 sites overlapped with SHP sites (data not shown).

Since the role of SREBP-2 in regulating the sterol biosynthetic pathway is well known, we compared binding of SHP and SREBP-2 near to genes in this pathway. Strikingly, SREBP-2 binding coincident with SHP binding was detected at genes related with sterol biosynthesis, including the rate-limiting enzyme HMGCR, a master transcription activator, SREBP-2, and its partner proteins, INSIG and SCAP (Fig. 4c, Additional file 1: Figure S3). In GO analysis of genes with overlapping peaks for SHP and SREBP-2, a number of pathways related with metabolic processes were enriched (Additional file 1: Figure S4). These genomic analyses suggest a potential partnership of SHP and SREBP-2 in regulation of sterol biosynthetic genes in the liver.

### SHP is recruited to SREBP-2 target sterol biosynthesis-related genes and inhibits their expression

**Fig. 5:**
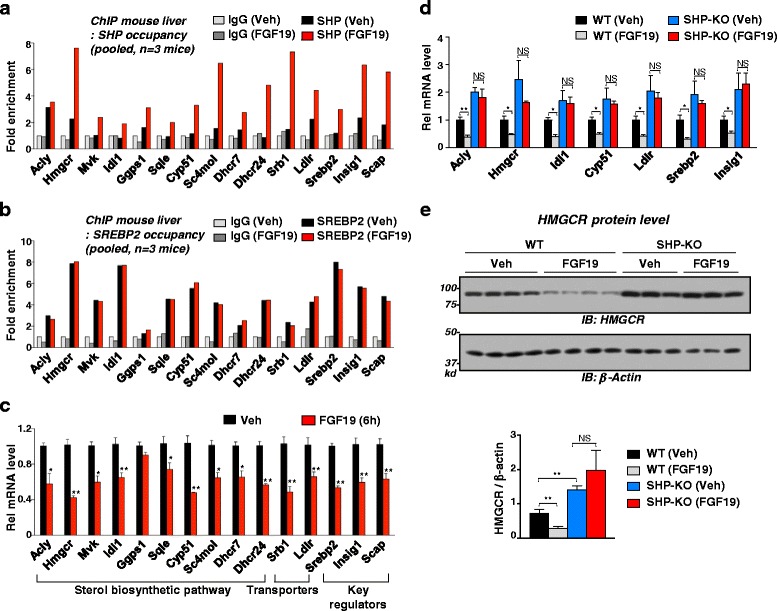
SHP was recruited to SREBP-2 target sterol biosynthesis-related genes and inhibits their expression. Mice were treated with vehicle or FGF19 and livers were pooled from three mice for ChIP assays (**a, b**) to examine occupancy of SHP (**a**) and SREBP-2 (**b**) at the indicated genes, and mRNA levels of genes near SHP binding peaks (**c**). Statistical significance was determined by the Student’s *t*-test (SEM, n = 3, *P <0.05, **P <0.01). **d, e** WT or SHP-KO mice were treated with vehicle or FGF19 for 6 h and livers were collected. **d** Effects of FGF19 on mRNA levels of indicated genes. **e** Hepatic protein levels of HMGCR in WT and SHP-KO mice were measured and relative HMGCR levels are shown (right)

We next examined the effect of FGF19 on the occupancy of SHP and SREBP-2 at cholesterol biosynthesis-related genes. Occupancy of SHP was increased, while occupancy of SREBP2 was detected but not increased, by FGF19 at nearly all genes tested in WT mouse liver (Fig. 5a, b) and in primary mouse hepatocytes and increased SHP binding was not detected in SHP-KO mice (Additional file 1: Figure S5a, b). Consistent with the co-repressor function of SHP [13, 23, 24], increased occupancy correlated with decreased mRNA levels by FGF19 treatment (Fig. 5c). FGF19 inhibition of expression of these genes was not observed in SHP-KO mice (Fig. 5d) and protein levels of the rate-limiting enzyme for sterol biosynthetic pathway, HMGCR, was also decreased by FGF19 in a SHP-dependent manner (Fig. 5e). These results suggest that SHP is recruited to SREBP-2 target genes related with cholesterol biosynthesis and inhibits these genes.

### FGF19 increased the functional interaction between SHP and SREBP-2 at *Hmgcr*, resulting in epigenomic repression

**Fig. 6:**
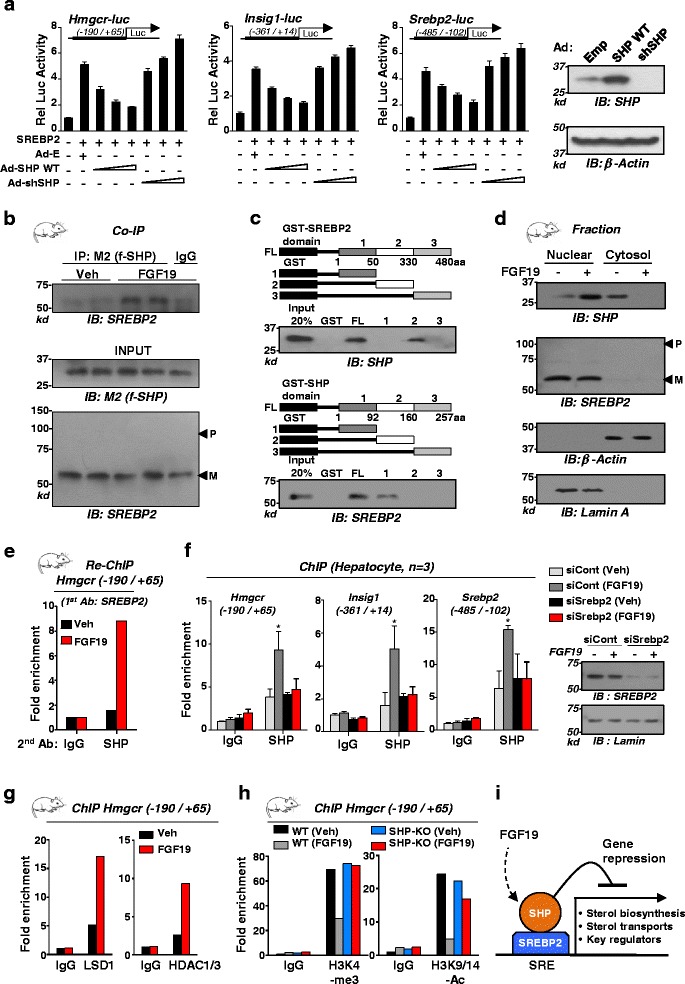
FGF19 increased functional interaction of SHP with SREBP-2, resulting in epigenomic repression. **a** Reporter assay: HepG2 cells were transfected with plasmids and infected with adenoviruses as indicated, treated with FGF19, and luciferase activity was normalized to β-galactosidase activity. Protein levels of SHP are shown at right. **b** CoIP: Mice were tail vein injected with Ad-flag-SHP, 1 week after infection, mice were treated with vehicle or FGF19 for 2 h, and CoIP assays using liver nuclear extracts were performed. P or M indicates the precursor or mature form of SREBP-2, respectively. **c** GST pull-down: Schematics of the SHP and SREBP-2 domains fused to GST are shown at the top. The amount of SHP and SREBP-2 bound to the reciprocal GST-proteins were determined by IB. **d** Fractionation study: Effects of FGF19 on protein levels of SHP and SREBP-2 in nuclear and cytoplasmic fractions of mouse liver extracts. Actin and lamin were markers for the cytoplasm and nucleus, respectively. **e** Liver re-ChIP: Mice were treated with vehicle or FGF19 for 2 h and livers were pooled from three mice. Chromatin was immunoprecipitated with SREBP-2 antibody first and then eluted and re-precipitated with SHP antibody. **f** siRNA/ChIP: Hepatocytes were transfected with siRNA for SREBP-2 or control RNA and then 3 days later, cells were treated with vehicle or FGF19 for 2 h and ChIP assays were done. Statistical significance was determined by the Student’s *t*-test (SEM, n = 3, *P <0.05). At right, levels of nuclear SREBP-2 protein levels detected by IB are shown. **g**, **h** ChIP: WT or SHP-KO mice (pooled from three mice) were treated with vehicle or FGF19 and ChIP assays were done. **i** Model illustrating molecular mechanisms by which SHP inhibits SREBP-2 direct target genes

To test the effect of SHP on SREBP-2 activity, DNA regions of *Hmgcr*, *Insig-1*, and *Srebp-2* containing both SHP and SREBP-2 binding sites*,* were cloned into luciferase plasmids for reporter assays. Expression of SREBP-2 increased luciferase activities, and co-expression of SHP inhibited the increased transactivation, while downregulation of SHP enhanced the increase (Fig. 6a). These results suggest that SHP inhibits SREBP-2 activation of these genes.

To further examine the effect of FGF19 on SHP regulation of SREBP-2 activity, we performed CoIP protein interaction studies. FGF19 treatment increased the interaction of adenovirally expressed SHP with SREBP-2 in liver extracts (Fig. 6b). In GST pull-down studies using partially purified proteins (Additional file 1: Figure S6), full length SREBP-2 and its central domain fragment (50-330 aa) bound to SHP, while full length SHP and its N-terminal domain (1-92 aa) bound to SREBP-2 (Fig. 6c), indicating that SHP and SREBP-2 directly interact.

FGF19 treatment increased nuclear localization of SHP in the nucleus (Fig. 6d). This observation together with the observations of direct interaction between SHP and SREBP-2, which was increased interaction by FGF19, suggests that SREBP-2 may recruit SHP to its target cholesterol biosynthetic genes and that these two proteins co-occupy the same genomic site in these genes. We, thus, examined these possibilities using *Hmgcr* gene as a model by re-ChIP assays. In chromatin first precipitated by SREBP-2 antibody, *Hmgcr* sequence enriched by re-precipitation with SHP antibody was increased by FGF19 (Fig. 6e). Since occupancy of SREBP-2 at *Hmgcr* was not increased by FGF19 (Fig. 5b), these results suggest that SREBP-2 binds constitutively to *Hmgcr* and recruits SHP to the gene upon FGF19 treatment. Increased SHP occupancy at *Hmgcr*, *Insig-1*, and *Srebp-2* by FGF19 treatment was nearly abolished when SREBP-2 was downregulated in hepatocytes (Fig. 6f), indicating that SHP occupancy is dependent on SREBP-2.

SHP inhibits its target genes by recruiting repressive histone modifiers, such as HDACs and LSD1, resulting in epigenomic repression [23–25]. As expected, occupancy of LSD1 and HDAC1/3 was increased by FGF19 (Fig. 6g), and levels of H3K4-me3 and H3K9/14-Ac, gene activation histone markers [26], were decreased by FGF19 but these effects were attenuated in SHP-KO mice (Fig. 6h). As illustrated in Fig. 6i, these results suggest that SHP is recruited to SREBP2-bound chromatin at sterol biosynthetic genes by FGF19 treatment, resulting in epigenomic repression.

### Feeding inhibited SREBP-2 target sterol biosynthetic genes in a SHP-dependent manner

After a meal, FGF15/19 is induced by BA-activated FXR in the ileum and acts at the liver to mediate postprandial responses [14, 15]. In humans, serum FGF19 levels were shown to be elevated about 3 h after feeding long after insulin levels are increased [27]. We, therefore, examined the effects of feeding, which would activate endogenous FGF15 signaling by BA-activated intestinal FXR on the functional interaction of endogenous SHP and SREBP-2.

**Fig. 7:**
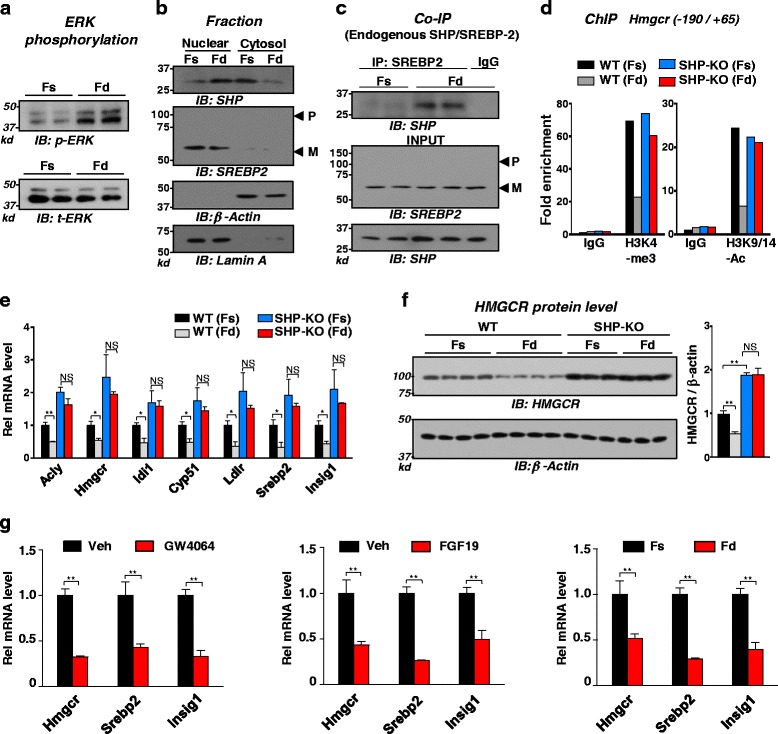
Feeding inhibited SREBP-2 target sterol biosynthesis genes in a SHP-dependent manner. WT or SHP-KO mice were fasted for 12 h and then fed or fasted for 6 h and livers were collected. **a** Levels of phosphorylated ERK and total ERK levels are shown. **b** Fractionation study: Protein levels of SHP and SREBP-2 in nuclear and cytoplasmic fractions of mouse liver extracts were measured. **c** CoIP assays were done to monitor effect of feeding on interaction with endogenous SHP and SREBP-2. **d**-**f** ChIP: Effects of feeding and fasting in WT or SHP-KO mice on (**d**) gene activation histone marks at *Hmgcr* gene, (**e**) the mRNA levels of indicated genes, and (**f**) hepatic protein levels of HMGCR. Statistical significance was determined by the Student’s *t*-test, (SEM, n = 3-4, *P <0.05, **P <0.01, and NS, statistically not significant). **g** Mice were fasted for 12 h and then, treated with GW4064, FGF19, or feeding for 6 h and livers were collected to measure mRNA levels of *Hmgcr*, *Srebp2*, and *Insig1* genes by q-RTPCR. Statistical significance was determined by the Student’s *t*-test, (SEM, n = 5 mice, *P <0.05, **P <0.01)

Increased levels of phosphorylated ERK, a marker of FGF15/19 signaling [14, 15, 28], were detected in mice fed for 6 h after 12 h of fasting (Fig. 7a). In cellular fractionation studies, feeding increased nuclear abundance of SHP, but SREBP-2 was constitutively localized in the nucleus (Fig. 7b). Importantly, the interaction in liver nuclear extracts between endogenous SHP and SREBP-2 was increased by feeding (Fig. 7c). In ChIP assays, feeding decreased levels of gene activation histone marks, H3K4-me3 and H3K9/14-Ac, at *Hmgcr* gene in WT mice, but these effects were diminished in SHP-KO mice (Fig. 7d). Feeding also inhibited expression of direct SREBP-2 target sterol biosynthesis-related genes (Fig. 7e) and significantly decreased HMGCR protein levels in WT mice (Fig. 7f). In sharp contrast, in SHP-KO mice, basal expression of these genes in fasted mice was highly elevated and was not decreased by feeding nor were HMGCR protein levels decreased. Treatment with an FXR agonist, GW4064, which would result in intestinal induction of endogenous FGF15, also decreased expression of these genes similar to those treated with FGF19 or feeding (Fig. 7g). These results suggest that the functional interaction between endogenous SHP and SREBP-2 is increased in the nucleus physiologically by feeding and that SHP is important for feeding-mediated inhibition of SREBP-2 target genes involved in sterol biosynthesis.

### FGF19-induced phosphorylation of SHP at Thr-55 is important for inhibition of sterol biosynthetic genes and reducing cholesterol levels

**Fig. 8:**
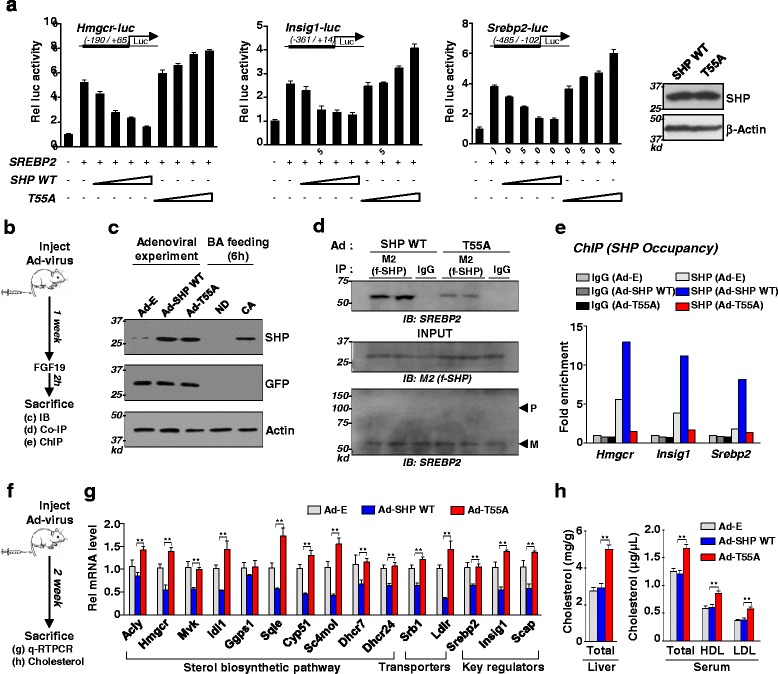
Role of FGF19-induced phosphorylation of SHP at Thr-55 in functional interaction with SREBP-2 and inhibition of sterol biosynthetic genes. **a** Luciferase reporter assay: HepG2 cells were transfected with the plasmids indicated, treated with FGF19, and luciferase/β-galactosidase activities were measured. **b** Experimental outline for adenoviral experiments. **c** Mice were injected via the tail vein with control Ad-empty, Ad-SHP WT, or Ad-T55A and then 2 weeks later, livers were collected. For BA feeding (0.5 % CA diet) experiments, mice were fed normal diet (ND) or CA for 6 h and then, livers were collected. Liver extracts (pooled from n = 3) were prepared and protein levels of SHP, GFP, and actin were detected by IB. **d** Mice were injected via the tail vein with adenovirus and 1 week later, the injected mice were treated with vehicle or FGF19 for 2 h and CoIP assays were done in liver extracts and (**e**) ChIP assays were done using livers pooled from three mice. **f** Experimental outline. Mice were injected with Ad-SHP WT or the T55A mutant, and 2 weeks later, (**g**) mRNA levels of indicated genes and (**h**) liver and serum cholesterol levels were measured

An important question is how SHP senses the FGF19 signal to mediate transcriptional responses. It was shown that SHP is phosphorylated at Thr-55 by PKCζ upon FGF19 signaling [13]. To test if this phosphorylation of SHP at Thr-55 is important for inhibition of SREBP-2 transactivation by FGF19, we examined the effects of mutation of Thr-55 on expression of *Hmgcr*, *Insig*, and *Srebp-2* genes using luciferase reporter plasmids containing the promoter regions of these genes that bound both SHP with SREBP-2. Expression of SREBP-2 increased the luciferase activity and overexpression of SHP wild type (WT) decreased the activity in a dose-dependent manner (Fig. 8a). In contrast, these effects were not observed with the phosphorylation-defective T55A mutant and notably, the T55A mutant acted like a dominant negative mutant and increased expression. These data suggest that FGF19-induced Thr-55 phosphorylation of SHP is important for its inhibition of SREBP-2 activity.

To determine the role of phosphorylation of SHP on functional interaction with SREBP-2 in the liver, mice were tail vein injected with adenovirus expressing SHP WT or T55A and 1 week later, the mice were treated with FGF19 for 2 h (Fig. 8b). The protein levels of SHP in adenoviral-mediated expression groups were similar to physiological levels of SHP after BA-mediated induction (Fig. 8c). In CoIP studies, the increased interaction of SHP with SREBP-2 by FGF19 was markedly diminished with the T55A mutant compared to control WT (Fig. 8d). In ChIP assays, the increased SHP occupancy at selected SREBP-2 target genes by FGF19 treatment was not observed with the T55A mutant (Fig. 8e). These results suggest a potential role of FGF19-induced phosphorylation of SHP in functional interaction with SREBP-2 in the nucleus.

Finally, we determined the effects of mutation of Thr-55 on expression of key sterol biosynthetic genes and serum/liver cholesterol levels. Adenoviral-mediated liver-specific expression of SHP WT (Fig. 8f) resulted in decreased mRNA levels of nearly all tested genes but these effects were completely blocked in mice expressing the phosphorylation-defective Thr-55 SHP mutant (Fig. 8g). As expected from the increased expression of cholesterol biosynthetic genes, levels of cholesterol in liver and serum were significantly increased in mice expressing the Thr-55A SHP mutant (Fig. 8h). These studies suggest that FGF19-induced phosphorylation of SHP at Thr-55 is important for its functional interaction with SREBP-2 and inhibition of SREBP-2 target genes related with the sterol biosynthetic pathway.

## Discussion

We have identified genome-wide binding sites of SHP in hepatic chromatin and demonstrate that SHP is a new transcriptional partner with SREBP-2 in the regulation of cholesterol biosynthesis. The ChIP-seq analysis revealed 1,508 and 3,155 binding sites in vehicle- and FGF19-treated groups, respectively, with about 685 sites present in both groups. The overall pattern of SHP binding between these two groups was similar but SHP binding was enhanced at the sites by FGF19. Remarkably, SHP binding peaks in both groups showed a strong preference for the promoter regions, which provides confidence that the binding is functionally significant. Potential new functions directly regulated by SHP were identified and unexpected motifs for non-nuclear receptor transcription factors that are enriched in SHP binding regions were also identified.

**Fig. 9:**
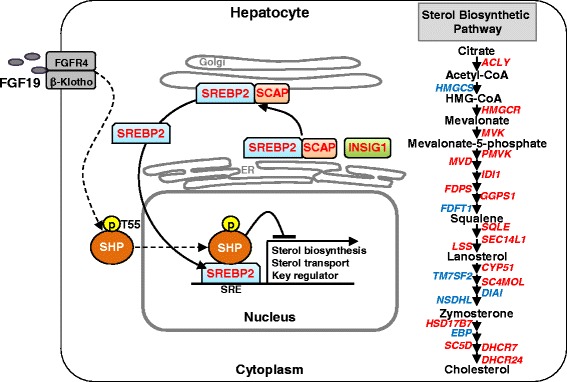
Model: Treatment with FGF19 or physiological activation of FGF15 in mice by feeding increases the interaction between endogenous SHP and SREBP-2 in the nucleus in the liver and inhibits expression of direct SREBP-2 targets in the sterol biosynthetic genes, in a SHP-dependent manner. FGF19-induced phosphorylation of SHP at Thr-55 appears to be important for functional interaction with SREBP-2 in the nucleus. Hepatic genes that have shared binding peaks of SHP and SREBP-2 identified by ChIP-seq, ChIP, or both are indicated in red

A striking finding is that SHP acts as a global partner with SREBP-2 in regulation of cholesterol biosynthesis. A remarkable global overlapping of many SHP binding sites with those of SREBP-2, previously published [20], was detected. Over 40 % of the SREBP-2 sites co-localized with SHP sites and about 20 % of the SHP sites were co-localized with SREBP-2 sites. Remarkably, SHP binding was detected at most of SREBP-2 target genes related with sterol biosynthesis, including *Hmgcr*. We showed that endogenous SHP interacts with SREBP-2 in response to FGF19 or feeding, is recruited to direct SREBP-2 target sterol biosynthetic genes, and inhibits their expression (Fig. 9). We further showed that FGF19 signal-induced phosphorylation of SHP at Thr-55 appears to be important for functional interaction with SREBP-2 in the nucleus. These molecular and biochemical studies, together with genomic analyses, demonstrate that SHP and SREBP-2 are transcriptional partners in the regulation of cholesterol biosynthesis gene networks in response to FGF19.

The functional role of SHP in the inhibition of the conversion of cholesterol to BAs to reduce liver BA levels and prevent hepatotoxicity has been established [5, 6, 10], but the consequential increase in cholesterol levels could detrimentally result in hypercholesterolemia and related diseases. Our present study suggests that SHP, with SREBP-2, inhibits expression of hepatic genes involved in sterol biosynthesis and regulatory processes, which would likely counteract the inhibition of cholesterol catabolism and prevent excessive accumulation of cholesterol. Strikingly, expression of nearly all tested genes in the cholesterol biosynthetic and transporter pathways, as well as key regulators, were inhibited by FGF19 or feeding, which importantly, was attenuated in SHP-KO mice, demonstrating a role for SHP in transcriptional inhibition of sterol biosynthetic gene networks during the postprandial period, which provides a potential mechanism for cholesterol-lowering action of FGF15/19 [16]. Our findings, together with the well-known function of SHP in suppressing BA biosynthetic genes, identify SHP as a global transcriptional regulator in maintaining interrelated cholesterol and BA homeostasis.

## Conclusion

Our genome-wide analysis of SHP binding sites in hepatic chromatin in mice treated with vehicle or FGF19 reveals unexpectedly that SHP functions as a global transcriptional partner of SREBP-2 in regulation of sterol biosynthetic gene networks. Pharmacological activation of the FGF19 pathway has attractive therapeutic potential for treatment with metabolic disease and bile acid-related hepatobiliary disorders [16, 29], and in fact, FGF19 analogs are currently being tested in clinical trials. For this reason, the present study, not only greatly increases our understanding of the global function of SHP in mediating FGF19 metabolic action, but also provides useful information for developing therapeutic strategies for FGF19-associated diseases.

## Methods

### Reagents

Antibodies for SHP (sc-30169), SREBP-2 (sc-5603), HDAC1 (sc-7872), HDAC3 (sc-11417), and Lamin A (sc-20680) were purchased from Santa Cruz Biotechnology; for M2 (F3165) from Sigma, for β-actin (#4970) from Cell Signaling, for LSD1 (ab17721) from Abcam, and H3K9/K14-Ac (#06-599) and H3K9-me2 (#07-030) from Millipore. SREBP-2 siRNA (M-050073-01) was purchased from Dharmacon, Inc., and pcDNA3.1-flag-SREBP-2 was obtained from Addgene.

### Animal experiments

Male C57BL6 mice or SHP-KO mice (8-12 weeks old) were fasted for 12 h and injected via the tail vein with vehicle or FGF19 (1 mg/kg) at 09:00, and 2 h or 6 h later, livers were collected. For adenoviral experiments, C57BL6 mice were injected via the tail vein with adenoviruses (0.5-1.0 × 10^9^ active viral particles in 100 μL PBS) and 1-2 weeks later, the mice were sacrificed as described [12, 13, 30]. For feeding or GW4064 experiments, mice were fasted for 12 h and then, fed for 6 h or treated with GW4064 (30 mg/kg in corn oil) for 6 h, and livers were collected for further analyses.

### Ethical approval

All animal use and adenoviral protocols were approved by the Institutional Animal Care and Use and Institutional Biosafety Committees and were in accordance with National Institutes of Health guidelines.

### ChIP assays and genomic sequencing

Mice were fasted overnight and injected with vehicle or FGF19, livers were collected 2 h later, and ChIP assays were performed using SHP antibody (sc-30169). Three sets of input and immunoprecipitated samples from three mice were pooled and 18 ng DNA was used for genomic sequencing using the Illumina/Solexa Genome Analyzer II (Biotechnology Center, University of Illinois at Urbana-Champaign). To validate the specificity of the SHP antibody for ChIP-seq, SHP binding to hepatic genes was analyzed by three independent ChIP assays for vehicle- or FGF19-treated WT and SHP-KO mice (Additional file 1: Figures S1a, S5a).

### ChIP-seq mapping and peak finding

The raw ChIP-seq reads were mapped to the mouse reference genome (UCSC mm9) with up to two mismatches using Bowtie [31] and peaks were identified by MACS. Peaks with false discovery rate (FDR) <0.05 were kept. To estimate coverage of our data, a ChIP-seq saturation test was done by sub-sampling the reads from all mapped reads. The Pearson correlation coefficient was calculated for average reads coverage in 10,000 bp bins across the genome between the sub-sampled data and the full dataset using the bigwigCorrelate program from deepTools (version 1.5.11) [32]. We found that the correlation has reached 0.95 when 75 % of the reads were sampled. To determine the quality of the ChIP-seq data, quality metrics from the ENCODE project [33] were used as benchmarks including: ‘Number of unique mapped reads’, which was higher than the ENCODE mean; ‘Signal portion of tags’ (SPOT), with our samples substantially higher than the ENCODE median; ‘PCR bottleneck coefficient’, with our samples higher than at least 25 % of ENCODE data which is considered only ‘mild bottlenecking’; and ‘normalized strand cross-correlation coefficient’ and ‘relative strand cross-correlation coefficient’ which were not high, but mostly above 1 which is considered acceptable quality by ENCODE.

### Data availability

The primary ChIP-Seq data have been deposited to the NCBI GEO database under the following accession number, GSE74913.

### Peaks annotation and GO analysis

Genomic features associated with peaks were determined by CEAS [34]. For SHP peaks, the distance to nearest TSS and enriched GO terms (*P* value <1e-3, FDR <0.05) were reported by GREAT version 2.0.2 [35] (association rule: Single nearest gene: 50 kb max extension and curated regulatory domains included).

### Motif discovery

We used MEME-ChIP [36] for *de novo* identification of transcription factor motifs. The regions, +/- 100 bp centered on peak summits, were extracted and then input to MEME-ChIP. The top 20 motifs (motifs with E-values <0.05 were considered) with lengths ranging from 6 to 15 were compared to known motifs in database JASPAR_CORE_2014 [37]. Motifs were also compared with the results from the pipeline ‘peak-motifs’ [38] (motifs with E-values <0.01 were considered) which were in good agreement with the MEME-ChIP results (Additional file 2: Table S1).

### CoIP, Re-ChIP, and siRNA/ChIP

CoIP and re-ChIP assays were performed as described [13, 39, 40]. Briefly, in CoIP assays, mouse liver extracts were prepared in 50 mM Tris-HCl, pH. 8.0, 150 mM NaCl, 2 mM EDTA, 0.5 % NP40, 10 % glycerol. For siRNA/ChIP assays, hepatocytes were transfected with siRNA and 3 days later, ChIP assays were done as described [25, 30].

### Biochemical fractionation studies

Liver tissues were minced and then resuspended in hypotonic buffer and cells were lysed by homogenization. After centrifugation, the nuclear pellet and cytoplasmic supernatant were collected for IB.

### GST pull-down assays

DNA fragments containing SREBP-2 (amino acids, 1-480, 1-50, 51-330, and 331-480) were inserted into the pGEX4T-1 at BamH1/Xho1 sites. GST-fusion proteins were incubated with the reciprocal proteins that were synthesized by TNT (Promega, Inc.) and their interaction was detected by IB.

### Construction of *Hmgcr-luc*, *Insig1-luc*, and *Srebp2-luc*, and reporter assays

Genomic DNA fragments containing *Hmgcr* (-190/+65), *Insig-1* (-361/+14), or *Srebp-2* (-485/-102), were inserted into the pGL3-basic-luc plasmid and used for reporter assays.

### q-RTPCR

Total RNA was isolated, cDNA was synthesized, and q-RTPCR was performed and the amount of mRNA for each gene was normalized to that of 36B4. Primer sequences used in q-RTPCR and ChIP are listed in Additional file 2: Table S2.

## Additional files

Additional file 1: Figure S1. Information about the quality of SHP antibody for ChIP and ChIP-seq analysis. **Figure S2.** Validation of SHP binding from ChIP-seq analysis. **Figure S3.** Effects of FGF19 treatment on SHP binding at hepatic genes. **Figure S4.** Functional GO analysis of SREBP2 and SHP shared binding sites. **Figure S5.** Effects of FGF19 on SHP binding at sterol biosynthetic genes in WT and SHP-KO mice and in primary mouse hepatocytes. **Figure S6.** GST-SHP and GST-SREBP2 proteins used in GST pull-down assay. (PDF 801 kb) Additional file 2: Table S1. Comparison between MEME-ChIP and peak-motifs on predicting interacting TFs. **Table S2.** Primer sequences used in q-RTPCR and ChIP studies. (PDF 14 kb)
