# Supplementary material for: Liver ChIP-seq analysis in FGF19-treated mice reveals SHP as a global transcriptional partner of SREBP-2
Source: Genome Biol. 2015 Dec 4;16:268. doi: 10.1186/s13059-015-0835-6 (PMC4669652; doi:10.1186/s13059-015-0835-6)
Supplement: Additional file 1: Figure S1. — Information about the quality of SHP antibody for ChIP and ChIP-seq analysis. Figure S2. Validation of SHP binding from ChIP-seq analysis. Figure S3. Effects of FGF19 treatment on SHP binding at hepatic genes. Figure S4. Functional GO analysis of SREBP2 and SHP shared binding sites. Figure S5. Effects of FGF19 on SHP binding at sterol biosynthetic genes in WT and SHP-KO mice and in primary mouse hepatocytes. Figure S6. GST-SHP and GST-SREBP2 proteins used in GST pull-down assay. (PDF 801 kb) [file 13059_2015_835_MOESM1_ESM.pdf]

Figure. S1

a

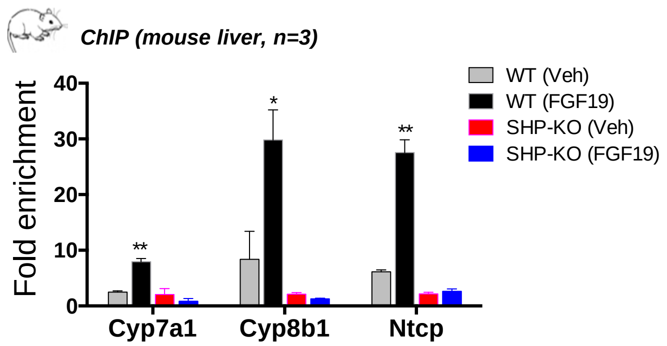

b

Treatment	Group	Unique Mapped Reads	Total Reads
Veh	Input	*16,743,814*	*22,029,930*
FGF19	Input	*17,849,172*	*23,247,293*
Veh	Shp	*17,968,561*	*32,763,382*
FGF19	Shp	*16,301,598*	*25,068,418*

Figure. S2

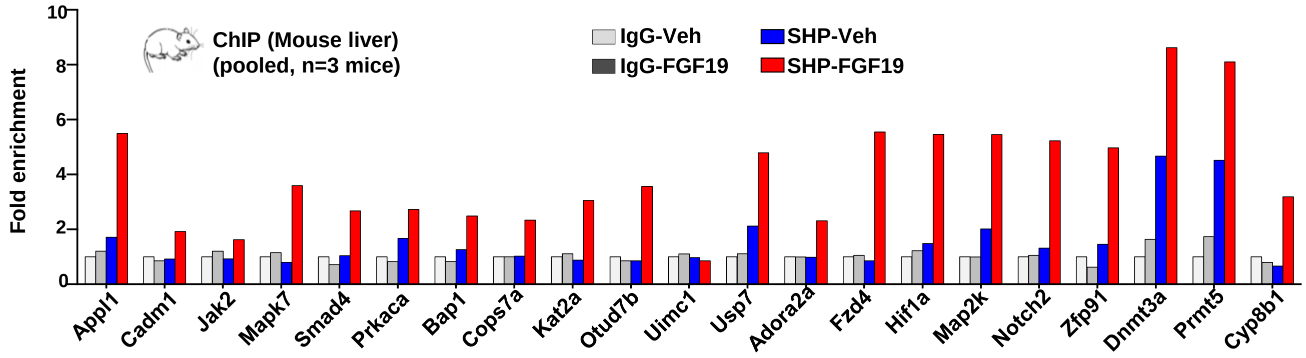

Figure. S3

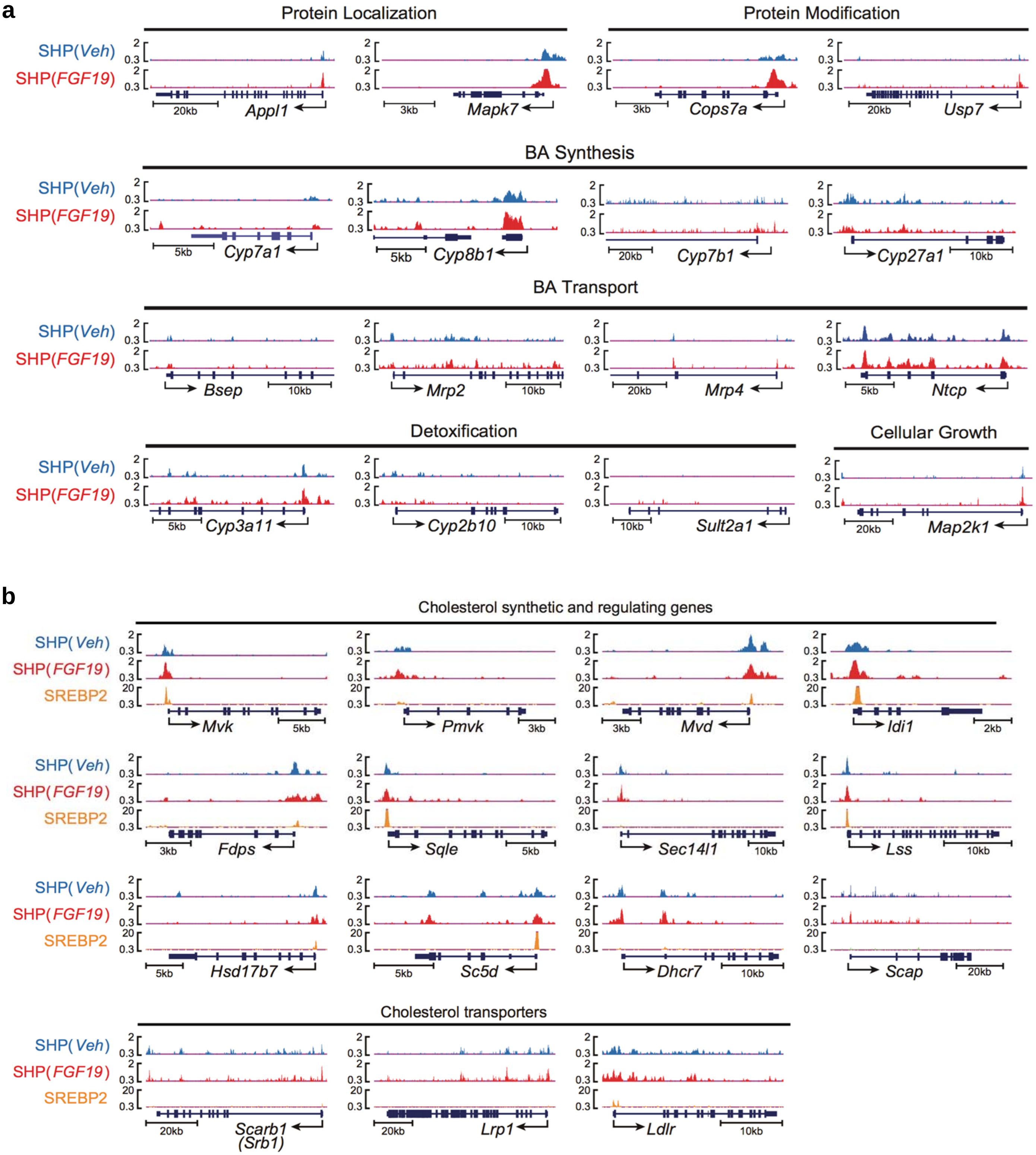
a
Protein Localization
Protein Modification
SHP(Veh)
SHP(FGF19)
Appl1
Mapk7
Cops7a
Usp7
20kb
3kb
BA Synthesis
Cyp7a1
Cyp8b1
Cyp7b1
Cyp27a1
5kb
10kb
BA Transport
Bsep
Mrp2
Mrp4
Ntcp
Detoxification
Cellular Growth
Cyp3a11
Cyp2b10
Sult2a1
Map2k1
b
Cholesterol synthetic and regulating genes
SREBP2
Mvk
Pmvk
Mvd
Idi1
2kb
Fdps
Sqle
Sec14l1
Lss
Hsd17b7
Sc5d
Dhcr7
Scap
Cholesterol transporters
Scarb1
(Srb1)
Lrp1
Ldlr

Figure. S4

GO analysis of SREBP2 and SHP (Veh / FGF19) shared binding

Gene Ontology Term	Gene count (Total)	P-value
cellular metabolic process	380 (6562)	5.15E-143
cellular macromolecule metabolic process	299 (4787)	4.40E-113
nitrogen compound metabolic process	222 (3803)	2.41E-78
regulation of cellular metabolic process	224 (3874)	2.63E-68
cellular biosynthetic process	186 (3016)	1.40E-65
RNA metabolic process	156 (2328)	4.83E-60
transcription, DNA-dependent	124 (1738)	4.74E-48
macromolecule modification	116 (1794)	4.83E-38
response to stimulus	220 (6440)	3.01E-34
developmental process	157 (3511)	1.27E-28
organelle organization	89 (1522)	1.35E-28
localization	141 (3226)	2.03E-26
multicellular organismal development	145 (3197)	3.23E-26
negative regulation of biological process	116 (2315)	8.17E-26
transport	119 (2638)	6.79E-24
cell cycle	55 (815)	8.65E-21

Figure. S5

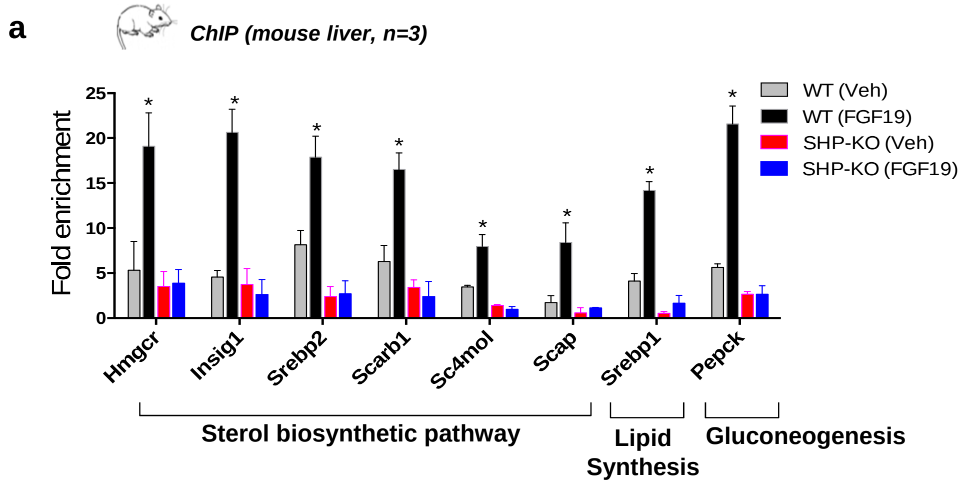

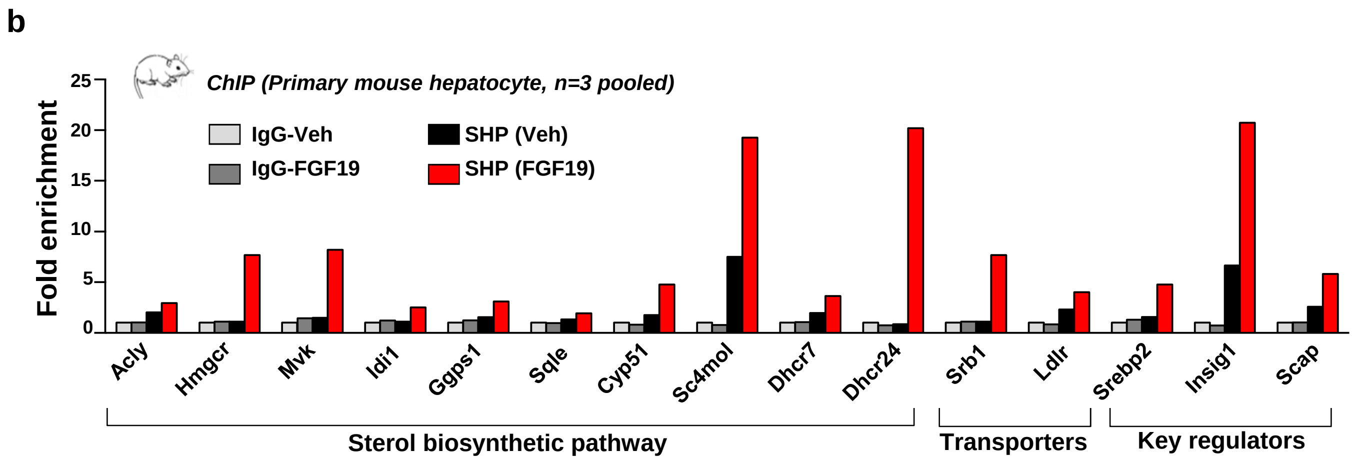

Figure. S6

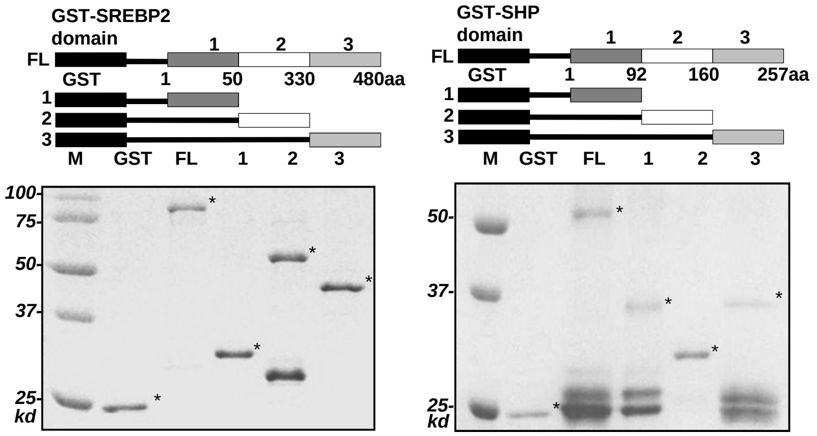

GST-SREBP2
domain
FL
1
2
3
GST
1
50
330
480aa
1
2
3
M
GST
FL
1
2
3
100-
75-
50-
37-
25-
kd
GST-SHP
domain
FL
1
2
3
GST
1
92
160
257aa
1
2
3
M
GST
FL
1
2
3
50-
37-
25-
kd
