# Supplementary material for: Liver ChIP-seq analysis in FGF19-treated mice reveals SHP as a global transcriptional partner of SREBP-2
Source: Genome Biol. 2015 Dec 4;16:268. doi: 10.1186/s13059-015-0835-6 (PMC4669652; doi:10.1186/s13059-015-0835-6)
Supplement: Additional file 2: Table S1. — Comparison between MEME-ChIP and peak-motifs on predicting interacting TFs. Table S2. Primer sequences used in q-RTPCR and ChIP studies. (PDF 14 kb) [file 13059_2015_835_MOESM2_ESM.pdf]

Table. S1 Comparison between MEME_ChIP and peak-motifs for prediction of interacting transcription factors

Veh		FGF19	
**MEME-ChIP**	**peak-motifs (RSAT)**	**MEME-ChIP**	**peak-motifs (RSAT)**
SP1/KLF5 (1e-131)	SP1/KLF5 (1e-046)	SP1/KLF5 (2.5e-256)	SP1/KLF5 (2.1e-068)
FOXP1 (6.1e-123)	FOXP1 (2.6e-007)	FOXP1 (5.8e-315)	FOXP1 (3.9e-010)
YY1/SRE (5.7e-095)	YY1/SRE (5.0e-017)	YY1/SRE (2.3e-131)	YY1/SRE (1.9e-024)
ZBTB33 (1.1e-068)	ZBTB33 (9.0e-013)	ZBTB33 (6.9e-025)	-
RUNX2 (2.6e-023)	-	RUNX2 (4.1e-059)	-
NRF1 (3.5e-017)	NRF1 (6.8e-015)	NRF1 (3.7e-067)	NRF1 (2.8e-026)
GABPA/ELK4 (2.3e-008)	GABPA/ELK4 (8.6e-009)	-	GABPA/ELK4 (2.8e-013)
USF1 (7.8e-006)	-	USF1 (1.1e-019)*	-
**-**	-	ZNF263 (2.4-150)	-
NFYA/NFYB (2.5e-002)*	NFYA/NFYB (8.5-009)	NFYA/NFYB (1.9e-016)*	NFYA/NFYB (9.1e-023)
**-**	RFX1 (9e-004)	RFX1 (5.6e-015)*	-

Table. S2

a List of mouse gene primer sequences for ChIP q-PCR analysis

Definition	Sense Primer	Anti-sense Primer
Appl1	TTCAATGGGCAGCTTGTCGA	TCGACATCCCCACACTGCT
Cadm1	ATATTTGCATAGGCCCCGC	AAAGGAGCAACAGCAGGAG
Jak2	AAGCTGAGGCAGAGCAGAAA	GGCTAAGGGGAAAGCAGGA
Mapk7	AAAACCGCGCCCTTGTCAAT	GGAGGCTGCTAGTACGCAT
Smad4	GATCTCTCACTCTGTCCCT	CAACTCGAAATTCCCGCCT
Prkaca	GACAGAGGACTTGGGCTGA	CCGCACGCAGTGAAGATCA
Bap1	GCTCGTCGTGTGATGATGA	CCAGCCAGCCCTTATTCAT
Adora2a	GCTGGTTTCGAAGTCGCCT	TGGTTCGCTCAGACCTCCA
Cops7a	CATGGTGTCTACCGGACGA	GAGGTGGCAGGAAAGGCTT
Kat2a	AGGAGTCGAAGTTGGGGCA	GTATGGAAGGCCAAAGGATG
Otud7b	TCCCAGCTCCCGCTCAAAA	GAGCTGGCCTCTTCCCAAT
Uimc1	AAGCTCGGATAGCAAGAGAG	CAGGAACTGTGTGAGCATAC
Usp7	GCTCGCTCTCAAAATGGC	TCCTTGTCCAGCCCAGTT
Fzd4	CTTTCATTGGACAGTCGCGT	GAATGTGCGGGATATCAGAC
Hif1a	TCCTTCACCATTGGCTCTC	TTGTCAAGGCCTGGCTAGA
Map2k	CAGCCCCAGCTTGGAGAAA	GACCTGCGTGCAAGAACCT
Notch2	AACTTTCAGCCAAACTTCGG	AGAGACAGAGAGGGATGGT
Zfp91	CCTTCCTGCTCTGGCCTTT	TTCAGAGACTTTCATCCGGC
Dnmt3a	*CTGTCCCTACGACCAGTGC*	GCCTCTTCTCTCCGCCTTC
Prmt5	GTCGCCTTAACAACCAGAGC	GGAGTACCAGTCCCAACGAG
Cyp7a1	ATATGCACAGGACCATGATC	CTTTGGTAGGTGAGCTCTTC
Cyp8b1	AAGCATGGGGATGTGTTCAC	CAAACTTGCGGAACTCCATG
Acly	CAGGAAGCTTAGTGGGTACT	CAGCCTCGTAGGTTCTCCT
Hmgcr	CTCGGCTCGGAGACCAATA	GCGGAAGGAACTGCGCTTA
Mvk	AGCACGTCATCGGTGTGAT	TGGGAACTTCAAGCAAGCC
Idi1	ACTGTAGTTGTCAGAGCGC	CGCCCTCTAACAGCATATC
Ggps1	CTCACGGTTCCACGATGAT	GTAGTCCAGTCTTCTAGGC
Sqle	TCTAGCCCGTGATCATCCA	GTAACCAGAGTAACCGCTG
Cyp51	TAGACGAGGCTGAGAGTGAA	GCGATCATTTGTGCAGCCAT
Sc4mol	CTCGCCTGTCGTAATTGGT	GGGTTCTGGATGAAGCCAA
Dhcr7	TCATAGCCCAGCTTCTGCA	AGCCTTCAAGCAAGCCAGT
Dhcr24	TGCTCTTTCTGCTCTGGGT	TTTCTAGGCACTGCGCATG
Srb1	TGAAGGAGACGGAGACAGA	ACCAGCAACCACCAGGAAA
Ldlr	AGCTTCAGGGGTTAAAAGAG	CGGTGCTCATCCTTAGCTT
Srebp2	AGCCTATCCCAGAATGTTAG	TGTTGTCAATGGGACCAGG
Insig1	GGATAGGCTGGGTGAGGTCA	TGCATCCTGTGGTTCCCGCT
Scap	AGGAGTTGAGAGGTGAAGG	AACCCCAAAGCAGGATCCA

b List of mouse gene primer sequences for q-RTPCR analysis

Definition	Sense Primer	Anti-sense Primer
36B4	CGACATCACAGAGCAGGC	CACCGAGGCAACAGTTGG
Acly	TGTTGACATTGGAGCCCTC	GATACAGCCCTTGCTTCAG
Hmgcr	TGTCCTTGATGGCAGCCTT	AAGCTGCCTTCTTGGTGCA
Mvk	AAGCCAGGTCTAGAGCAAG	TGGTCTCCCAGCAGTCAAA
Idi1	TGAAAGCCGAGTTGGGAAT	ATGTTCACCCCAGATACCA
Ggps1	AATCACTGGCTGAAAGTTCC	AACCACGTCGGAGCTTTGA
Sqle	TAAACTTGGTGGAGAGTGTG	CGTAGATTGCAACGGAAAAG
Cyp51	TTACAGGATAACCCAGCATC	GCATAGTCGACCAAATTGTC
Sc4mol	ATCACGTAATTCTCCTGTGG	GTGGAAATCATGGTGCCGA
Dhcr7	CCTACCTCTACACACTGCA	TAGTAACCCACGAGGCCAA
Dhcr24	AGCCACGTGTGAAGCACTT	AGCCATCGAACATCTCCCA
Srb1	TGCTGTGGTTCGAACAGAGC	GCCTGAATGGCCTCCTTATC
Ldlr	GCCCCAACAAGTTCAAGTGT	TGGTCTTGCACTCCTTGATG
Srebp2	GAACACCAAGCATGGAGAG	GACAGGAAAGAGAGGGGAA
Insig1	GAGGTGTCACAGTGGGAAA	CCACTCTGAACCATGTGCT
Scap	AGCCAAGACCATACTCTAAG	TCCATCTTGTCCTCCACTG
